# Recent progress on the role of miR-140 in cartilage matrix remodelling and its implications for osteoarthritis treatment

**DOI:** 10.1186/s13075-020-02290-0

**Published:** 2020-08-18

**Authors:** Li Duan, Yujie Liang, Xiao Xu, Yin Xiao, Daping Wang

**Affiliations:** 1 Department of Orthopedics, Shenzhen Intelligent Orthopaedics and Biomedical Innovation Platform, Guangdong Artificial Intelligence Biomedical Innovation Platform, Shenzhen Second People’s Hospital, The First Affiliated Hospital of Shenzhen University Health Science Center, Shenzhen, 518035 China; 2 Department of Child and Adolescent Psychiatry, Shenzhen Kangning Hospital, Shenzhen Mental Health Center, Shenzhen, 518003 China; 3 Institute of Health and Biomedical Innovation, Faculty of Science and Engineering, Queensland University of Technology, Kelvin Grove Campus, Brisbane, QLD 4059 Australia; 4 Department of Biomedical Engineering, Southern University of Science and Technology, Shenzhen, 518055 China

**Keywords:** Osteoarthritis, Cartilage matrix, Anabolism, Catabolism, miR-140

## Abstract

Cartilage matrix remodelling homeostasis is a crucial factor in maintaining cartilage integrity. Loss of cartilage integrity is a typical characteristic of osteoarthritis (OA). Strategies aimed at maintaining cartilage integrity have attracted considerable attention in the OA research field. Recently, a series of studies have suggested dual functions of microRNA-140 (miR-140) in cartilage matrix remodelling. Here, we discuss the significance of miR-140 in promoting cartilage formation and inhibiting degeneration. Additionally, we focused on the role of miR-140 in the chondrogenesis of mesenchymal stem cells (MSCs). Of note, we carefully reviewed recent advances in MSC exosomes for miRNA delivery in OA treatment.

## Background

Osteoarthritis (OA) is a prevalent joint disease, and the main characteristic of OA is cartilage degeneration. As the absolute number and percentage of the ageing population grows, OA will become a leading cause of disability [1]. Articular hyaline cartilage is a resilient and smooth elastic tissue that covers and protects the ends of long bones at the joints. The primary function of hyaline cartilage is to reduce friction and act as a cushion between joints. The dynamic balance of cartilage matrix metabolism is a crucial factor in maintaining joint integrity and movement [2]. Imbalance towards degradation of cartilage tissue causes a painful reaction and restricts joint mobility, ultimately resulting in OA [3]. A deep understanding of mechanisms in cartilage matrix metabolism will contribute to novel strategies for OA management. The role of microRNAs (miRNAs) in the pathology of diseases, such as miR-140 in OA, has attracted increasing interest. The safety and efficacy of current in vivo miR-140 delivery systems still need to be improved before their clinical application. Mesenchymal stem cell (MSC)-derived exosomes are a promising nanoscale system that could deliver miR-140 to treat OA in response to several vital questions.

## The loss of balance in cartilage matrix remodelling

Cartilage is a specialized connective tissue without blood or lymphatic vessels that are mainly composed of collagen type II (COL2) and proteoglycan. Chondrocytes are stable, postmitotic, differentiated cells and are the only cell type found in the cartilage matrix [4]. These cells are the primary regulators of matrix anabolism and catabolism in articular cartilage [5, 6]. Due to the previously mentioned characteristics, the cartilage matrix shows very low turnover in healthy adults. However, inductive stimuli, such as abnormal mechanical loading, increase the expression or activity of cartilage-degrading proteinases, which are primarily produced by chondrocytes [7]. With increased catabolic metabolism, the balance of cartilage matrix homeostasis is disturbed, resulting in OA [8].

### Cartilage matrix anabolism

Healthy adult articular cartilage contains approximately 5% of its volume as chondrocytes and 95% as extracellular matrix (ECM) [9]. The ECM is composed of a network of COL2 and an interlocking mesh of fibrous proteins and proteoglycans, hyaluronic acid, and chondroitin sulphate. Finite interactions between the ECM and chondrocytes maintain articular cartilage structural integrity and biological activity [4, 10]. The ECM regulates chondrocyte function via cell-matrix interactions, cytoskeleton organization, and integrin-mediated signalling. A disintegrin and metalloproteinases (ADAMs) in the cartilage matrix, such as ADAM12, regulate chondrocyte differentiation and proliferation [11]. Additionally, the ECM has a significant effect on the swelling behaviour and osmotic environment of chondrocytes [12].

On the other hand, factors produced by chondrocytes affect the synthesis of ECM [4]. Hyaline chondrocytes in healthy cartilage mainly secrete COL2. Chondrocytes also produce pro-collagen N-proteinases, such as a disintegrin and metalloproteinase with thrombospondin motifs (ADAMTS)-3, which is responsible for removing the N-terminal pro-peptide of type II pro-collagen and collagen fibril formation, thereby enhancing cartilage matrix anabolism [13]. However, increasing evidence shows that chondrocytes acquire a variety of degenerated phenotypes at the onset of OA. These cells can present a “dedifferentiated-like” phenotype, producing fibroblastic collagen type I (COL1) and collagen type III (COL3). Additionally, chondrocytes can acquire a hypertrophic phenotype and produce aberrant collagen type X (COL10) and catabolic matrix metalloproteinases (MMP)-13 [14]. In contrast to hyaline cartilage, hypertrophy and fibrocartilage eventually result in cartilage breakdown, thus stimulating OA development and joint dysfunction.

Promoting cartilage matrix anabolism is a feasible strategy to treat OA by maintaining cartilage integrity. Clinical studies have suggested that autologous chondrocyte implantation (ACI) is an effective way to repair cartilage defects, and it is the only cell-based therapy approved for OA treatment by the Food and Drug Administration (FDA) [15]. Due to limitations in the clinical application of ACI, alternative cell sources for OA treatment have been extensively investigated. Increasing evidence [16] suggests that MSCs have significant therapeutic potential for cell-based articular cartilage repair in patients with OA due to their ability to differentiate into chondrocytes and their paracrine regulation.

### Cartilage matrix catabolism

Cartilage matrix catabolism is a crucial factor in maintaining the dynamic balance of cartilage matrix metabolism. The increased degradation of cartilage collagen and the proteoglycan aggrecan (ACAN) principally leads to the degeneration of articular cartilage in arthritic joint diseases. Of the proteases that degrade cartilage collagens and proteoglycans, MMPs and aggrecanases have received the greatest attention [17]. Collagenases, such as MMP-2 and MMP-13 [18], have been detected in OA synovial fluids and joint tissues. Several studies have observed that aggrecanases, such as ADAMTS-4 and ADAMTS-5, are increased in OA cartilage [18, 19]. The results from in vitro and gene-modified animal studies suggest that these aggrecanases contribute to the degradation of cartilage matrix in OA [20, 21]. A fine-tuned balance exists between proteases and their inhibitors to maintain articular cartilage integrity. To date, a few inhibitors that target MMP-2 [22], MMP-13 [23, 24], ADAMTS-4 [25] and ADAMTS-5 [26] have been found to be effective in explants or rodent models. Several selective inhibitors targeting ADAMTS-4, and ADAMTS-5 have reached clinical trials in OA [27]. However, none of these treatments have proven to be effective. Thus, future studies should develop novel protease inhibitors.

## miR-140 in cartilage matrix remodelling

### miR-140

miRNAs are 20- to 23-nucleotide-long single-stranded noncoding RNA molecules that act as transcriptional repressors by binding to the untranslated region (UTR) of the target messenger RNA (mRNA) [28]. The primary function of miRNAs is to downregulate target gene expression [29]. More than 30 miRNAs that are expressed in human joint tissue are involved in cartilage homeostasis and OA development [30]. Among the known miRNAs, miR-140 has received increasing attention. miR-140 is located in one intron of the WW domain-containing E3 ubiquitin-protein ligase 2 (WWP2) gene [31]. The two products of RNA Dicer cleavage at the 5′ and 3′ end of the pre-miRNA, miR-140-3p and miR-140-5p, have been identified. miR-140 is evolutionarily conserved among vertebrates and is abundantly expressed in chondrocytes [32]. miR-140-deficient mice show age-related OA degenerative lesions, which are characterized by proteoglycan degradation and articular cartilage fibrosis. At 12 months, miR-140−/− mice show severe cartilage damage. However, miR-140 transgenic mice exhibit attenuated antigen-induced arthritis [33]. Thus, miR-140 is a regulator of cartilage homeostasis, and changes in its expression and functions play an important role in diseases associated with cartilage destruction.

### miR-140 targets

**Fig. 1:**
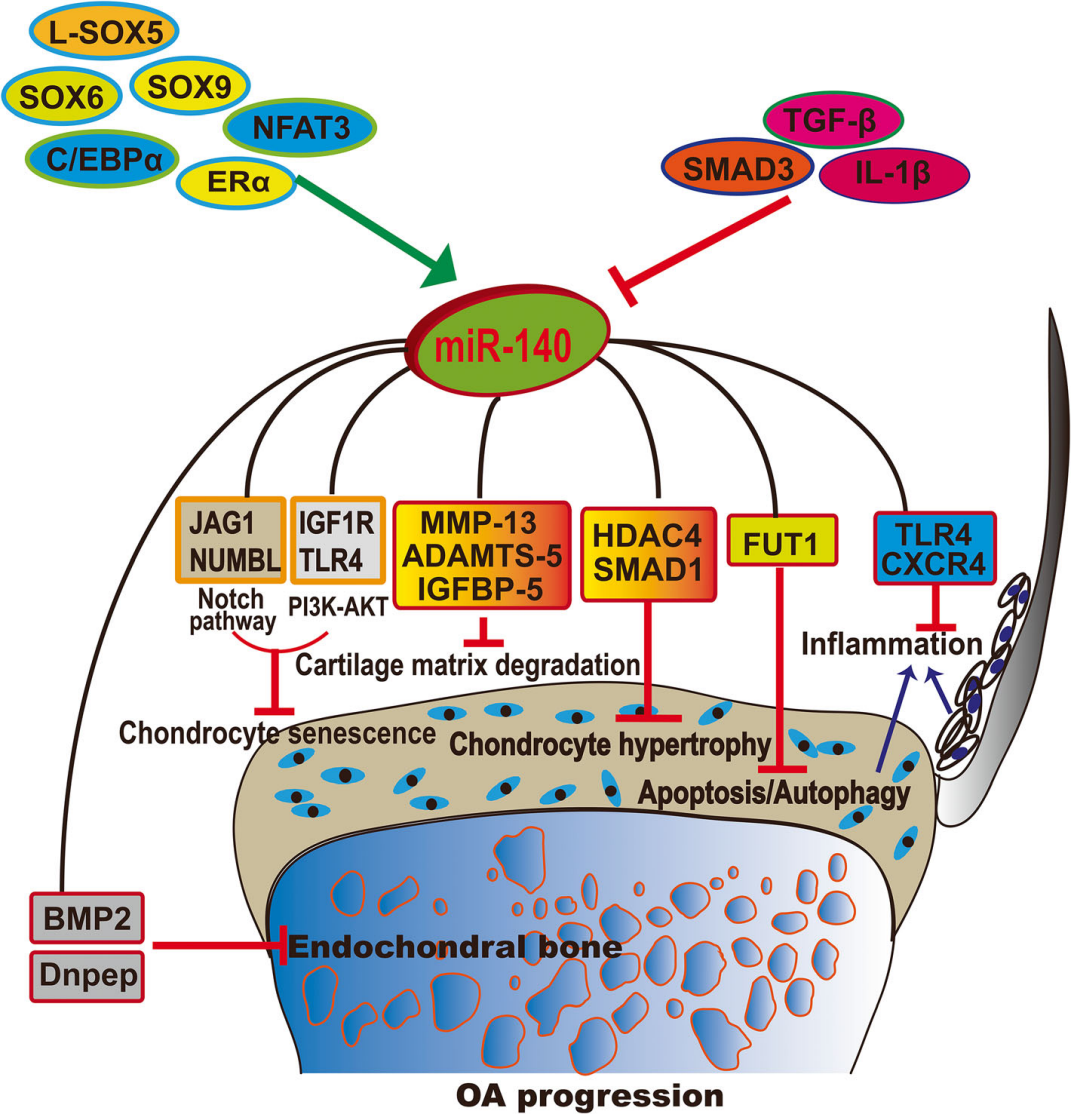
Upstream and target network of miR-140 involved in OA progression

Both miR-140-3p and miR-140-5p are expressed in the cartilage, and previous research has revealed that miR-140-3p is more abundant than miR-140-5p in human cartilage tissue [34]. The target genes of miR-140-5p and miR-140-3p differ, as summarized in Table 1 and illustrated in Fig. 1. To date, multiple targets of miR-140-5p have been identified as cartilage matrix modulators during the progression of OA from inflammation, chondrocyte hypertrophy and senescence to cartilage matrix degradation. miR-140-5p was found to inhibit inflammation by directly targeting Toll-like receptor (TLR)-4, C-X-C motif chemokine receptor (CXCR)-4, mothers against decapentaplegic homologue (SMAD) 3 [42], and interleukin-1 beta (IL-1β) [39]. Additionally, the inhibitory effect of miR-140-5p in chondrocyte hypertrophy has been shown through targeting histone deacetylase (HDAC)-4 [48] and SMAD1 [47]. During the inhibition of chondrocyte senescence, miR-140-5p targets Jagged1 (JAG1) and NUMB-like endocytic adaptor protein (NUMBL) in the Notch pathway and insulin-like growth factor 1 receptor (IGF1R) and TLR4 in the phosphatidylinositol 3′-kinase (PI3K)-AKT pathway [49]. Moreover, miR-140-5p inhibits cartilage matrix degradation through targets including MMP-13, ADAMTS-5, and insulin-like growth factor-binding protein (IGFBP)-5 [37]. Beyond miR-140 targets in the cartilage matrix, several studies have identified other targets in endochondral bone development, including bone morphogenic protein (BMP)-2 [38], DNPEP [32], and transforming growth factor beta receptor (TGFBR) 1 [40]. In comparison to the number of miR-140-5p targets, fewer miR-140-3p targets have been identified, including CXCR4, which is associated with in OA progression [35], and RAS-like proto-oncogene A (RALA), which is associated with MSC chondrogenesis [36].

**Table 1:** Function and targets of miR-140 in OA

miRNAs	Targets	Cell type/tissue	Function of miR-140	References
miR-140-3p	CXCR4	Chondrocytes	Ameliorates OA progression	[35]
	RALA	MSCs	Promotes chondrogenesis	[36]
miR-140-5p	IGFBP-5	OA chondrocytes	Inhibits OA progression	[37]
	BMP-2	Bone	Impairs embryonic bone development	[38]
	DNPEP	Bone	Promotes endochondral bone development	[32]
	IL-1β; IL-6; Syndecan-4	Chondrocytes	Inhibits mediators of inflammation and cartilage degradation and upregulates chondrogenic proteins	[39]
	TGFBR1	Stromal cell ST2 and preadipocyte 3 T3-L1	Promotes adipocyte differentiation and inhibits osteoblast differentiation from marrow stromal cells	[40]
	ADAMTS5	Chondrocytes	Inhibits ADAMTS5	[33]
	MMP-13	Chondrocytes	Inhibits IL-1β-induced MMP-13	[41]
	SMAD3	Mandibular condylar chondrocytes (MCCs)	Inhibits IL-1β-induced inflammation in MCCs	[42]
	HMGB1	Human chondrocytes C28/I2	Inhibits IL-1β induced OA cell model	[43]
	TLR4; BMP2	Adipose-derived MSCs	Inhibits osteogenesis	[44]
	RALA	MSCs	Promotes chondrogenesis	[36]
	FUT1	Chondrocytes	Inhibits apoptosis and promotes proliferation and autophagy of human primary chondrocytes	[45]
	HDAC4	Chondrocytes	Inhibits chondrocyte hypertrophy	[46]
	SMAD1	Chondrocytes	Inhibits chondrocyte hypertrophy	[47]

In the chondrogenic differentiation of MSCs and uncultured articular chondrocytes, miR-140-3p and miR-140-5p are both highly expressed. However, the expression of miR-140-3p and miR-140-5p is markedly and significantly downregulated in both chondrocytes and synovial fluid (SF) from knee OA [50]. The abundance of these miRNAs is inversely correlated with the severity of disease in knee OA [51]. Thus, miR-140-3p and miR-140-5p are both essential for chondrogenesis and cartilage homeostasis.

The transcription factors L-SOX5/SOX6/SOX9, C/EBP, NFAT3, and ERα upregulate miR-140 expression while TGF-ß/SMAD3 and IL-1ß downregulate miR-140 expression. miR-140 inhibits OA progression by (1) inhibiting chondrocyte senescence by targeting the Notch pathway (JAG1, NUMBL) and PI3K-AKT (IGF1R, TLR4), (2) inhibiting chondrocyte hypertrophy by targeting HDAC4 and SMAD1, (3) inhibiting chondrocyte apoptosis and autophagy by targeting FUT1, (4) inhibiting inflammation via targeting TLR4 and CXCR4, and (5) inhibiting cartilage matrix degradation by directly targeting MMP-13, ADAMTS-5, and IGFBP-5. For subchondral bone metabolism, miR-140 targets BMP-2 and DNPEP, thus inhibiting subchondral bone development.

### Upstream regulators of miR-140

Although WWP2 is the host gene of miR-140, different expression levels of WWP2 and miR-140 in OA chondrocytes have been detected, suggesting that other regulatory elements affect miR-140 expression independently of WWP2. Studies have identified the regulatory elements in the sequence upstream of pre-miR-140 (schematized in Fig. 1). For example, nuclear factor of activated T cells 3 (NFAT3) directly activates miR-140-5p expression in OA chondrocytes [52]. SRY-related high-mobility group box (SOX) 6 and L-SOX5 boost SOX9 to promote miR-140 expression during cartilage development [53]. Alternative activators of miR-140-5p include estrogen receptor (ER) and CCAAT/enhancer-binding proteins (C/EBPs). Previously, our research found that estrogen can upregulate miR-140-5p expression levels in IL-1β-induced OA-like chondrocytes since the miR-140 promoter contains ER response elements [41]. C/EBPα activates miR-140-5p expression in mesenchymal progenitor cell and induces adipogenesis [40]. By contrast, TGF-β/SMAD3 inhibits miR-140-5p expression in human OA chondrocytes [37, 52]. The role of hypermethylation of specific CpG sites in the miR-140 regulatory region is associated with decreased miR-140-5p expression in OA chondrocytes [54]. In addition, miR-140-5p expression was significantly decreased in chondrocytes following IL-1β stimulation.

### The role of miR-140 in cartilage matrix anabolism

SOX9, a high-mobility-group (HMG) domain-containing transcription factor, activates transcription by binding to a specific heptameric DNA sequence ((A/T)(A/T)CAA(A/T)G). As a pivotal transcription factor in chondrocytes, SOX9 regulates cartilage-specific genes, including COL2A1, COL9A1, COL11A1, ACAN, and others [55]. Existing research indicates that SOX9 is upstream of miR-140 in cartilage [56]. On the other hand, miR-140 targets RALA and stimulates in vitro chondrogenesis of bone marrow-derived MSCs (BM-MSCs) by increasing SOX9 and ACAN protein levels [36]. Thus, a bidirectional regulatory loop might exist between miR-140 and SOX9, thereby promoting cartilage matrix anabolism. In addition, miR-140 suppresses human chondrocyte hypertrophy by targeting SMAD1, which supports the anabolic gene expression of COL2A1 and inhibits the catabolic gene expression of COL10A1 and MMP-3 [47].

### The role of miR-140 in cartilage matrix catabolism

OA is a progressive disease of the joints that is characterized by degradation of articular cartilage and is mainly mediated by MMPs and ADAMTSs. To date, no effective treatment to terminate the gradual degeneration of the cartilage matrix has been developed. Thus, there is an urgent need to develop new strategies to target proteases. The synovial membrane shows signs of inflammation in OA patients, and synovial fibroblasts contribute to OA development by secreting inflammatory cytokines [57]. Therefore, strategies that can reduce both protease activity in the cartilage matrix and inflammation in synovial fibroblasts would be desirable. To detect the role of miR-140 in cartilage matrix degradation under OA pathological conditions, in vitro and in vivo experiments involving various genetic strategies in mice and rats were performed. These studies demonstrated that miR-140 in cartilage inhibits MMP-13 and ADAMTS-5 expression [41, 53]. miR-140-5p could also inhibit synovial fibroblast proliferation and secretion of IL-6 and IL-8 by regulating TLR4 expression [58]. Therapeutic interventions such as direct articular injection of miR-140 could influence the biological behaviour of synovial blasts and the subsequent inflammatory response of OA. Moreover, miR-140 attenuates the progression of early-stage OA by slowing chondrocyte senescence [49].

## miR-140 in mesenchymal stem cell chondrogenesis

### Mesenchymal stem cells in OA treatment

Unmodified MSCs or MSCs that have been modified using miRNA transduction are under active exploration as therapeutic agents [59, 60]. In contrast to autologous chondrocytes, MSCs are distributed in almost all tissues [61] and show increased potential for chondrogenic differentiation. Importantly, the therapeutic application of MSCs is promising because they possess extensive immunoregulatory properties via interaction with immune cells [62]. MSCs do not induce immunoreactivity or significant toxicity, even in xenogeneic recipients [63].

In the context of joint diseases such as OA, intra-articular administration of MSCs could be more beneficial than the intravenous/intraperitoneal route by applying the cells directly to the affected tissues. Preclinical models have detected the effect of MSCs in reducing cartilage degradation and joint inflammation. Ninety-nine clinical trials involving MSCs for OA treatment have been registered on ClinicalTrials.gov to date. Some results from clinical trials have provided initial evidence of the safety and efficacy of MSCs for OA treatment. However, the results of MSCs in OA therapy are quite inconsistent [64, 65]. The questions surrounding MSCs in OA treatment largely focus on their long-term therapeutic efficacy. More careful studies with randomized controls and larger sample sizes are required to estimate the potential of MSCs in cartilage repair and to evaluate the advantages and disadvantages of stem cell treatment.

### miR-140 promotes mesenchymal stem cell chondrogenesis

Some approaches have been implemented, such as overexpressing specific miRNAs, to improve the therapeutic effect of MSCs against OA. Building on the observation that miR-140 expression increased in parallel with SOX9 and COL2A1 expression during chondrogenic culture of MSCs [66], our group recently published that miR-140-transfected human umbilical cord (hUC)-MSCs stimulated cartilage repair in a rat model of knee OA [67], which is consistent with the in vivo protective effect of overexpressing miR-140-5p in SF-MSCs [68]. Additionally, miR-140-5p suppresses BMP2-mediated osteogenesis in undifferentiated human MSCs. Knockdown of miR-140-5p in MSCs can enhance MSC osteogenesis and promote fracture healing in an atrophic nonunion rat model [44].

## Exosomal delivery of miR-140 in OA treatment

### Exosomes

Exosomes are of endosomal origin and range in size from 40~160 nm in diameter. Almost all cell types secrete exosomes into the extracellular environment under both physiological and pathological conditions [69]. Exosomes encapsulate various molecular constituents of their parent cells, including proteins [70] and nuclear acids [71]. Similar biological functions of MSC-derived exosomes and intact MSCs have been shown, such as tissue repair and inflammation suppression [72, 73]. After administration, MSCs disappear from the target tissue quickly. However, MSCs are still able to exhibit cartilage-protective and immunomodulatory effects. Exosomes may mediate this effect by acting as paracrine activity effectors for MSCs [74]. Exosomes offer a host of advantages compared to those of stem cells. As cell-free therapeutic agents, MSC-derived exosomes cannot reproduce and thus are considered safer than cell therapy. Additionally, exosomes can be safely stored. Remarkably, exosomes can take up a variety of cargo molecules and transport these molecules to neighbour or distant cells, thus serving as mediators for intercellular communication [75].

Recently, exosomes have gained increasing attention in the study and research of OA. Purified exosomes isolated from inflamed SF are functionally active in their ability to stimulate the release of the pro-inflammatory cytokines IL-6 and tumour necrosis factor-alpha (TNF-α) from M1 macrophages [76], which may contribute to the initiation and progression of OA. Moreover, exosomes from therapeutic cells have also shown enormous potential for cartilage repair in OA. For example, intra-articular administration of primary chondrocyte exosomes could restore mitochondrial dysfunction and switch the macrophage phenotype towards M2 [77]. These M2 macrophages can produce anti-inflammatory cytokines (e.g., IL-4 and IL-1 receptor antagonist (IL-1RA) [78] and TGF-β), which promote collagen expression, thus helping to repair damaged tissues, clear debris and restore cartilage matrix homeostasis [79, 80].

### miR-140 delivery by exosomes

Previous studies have demonstrated a protective role of miR-140 in OA development. However, unprotected miR-140 is easily degraded by endogenous nucleases, especially in the context of an inflammatory disease such as OA. The lack of a practical or safe method to deliver miR-140 is an obstacle for real progress towards its intended use as a clinically usable therapeutic. To protect miR-140 from degradation and maintain its integrity and activity within the OA joint and synovium, considerable efforts have been made to develop strategies to improve miRNA delivery efficiency, such as chitosan nanoparticles [81], viruses and liposomes. For miR-140 therapeutic applications, these carriers should protect the RNA from degradation and accurately deliver the RNA to the desired cells, while avoiding delivery to nontarget cells. Of note, these carriers should encourage efficient cellular uptake and release the miRNA within the target cells.

**Fig. 2:**
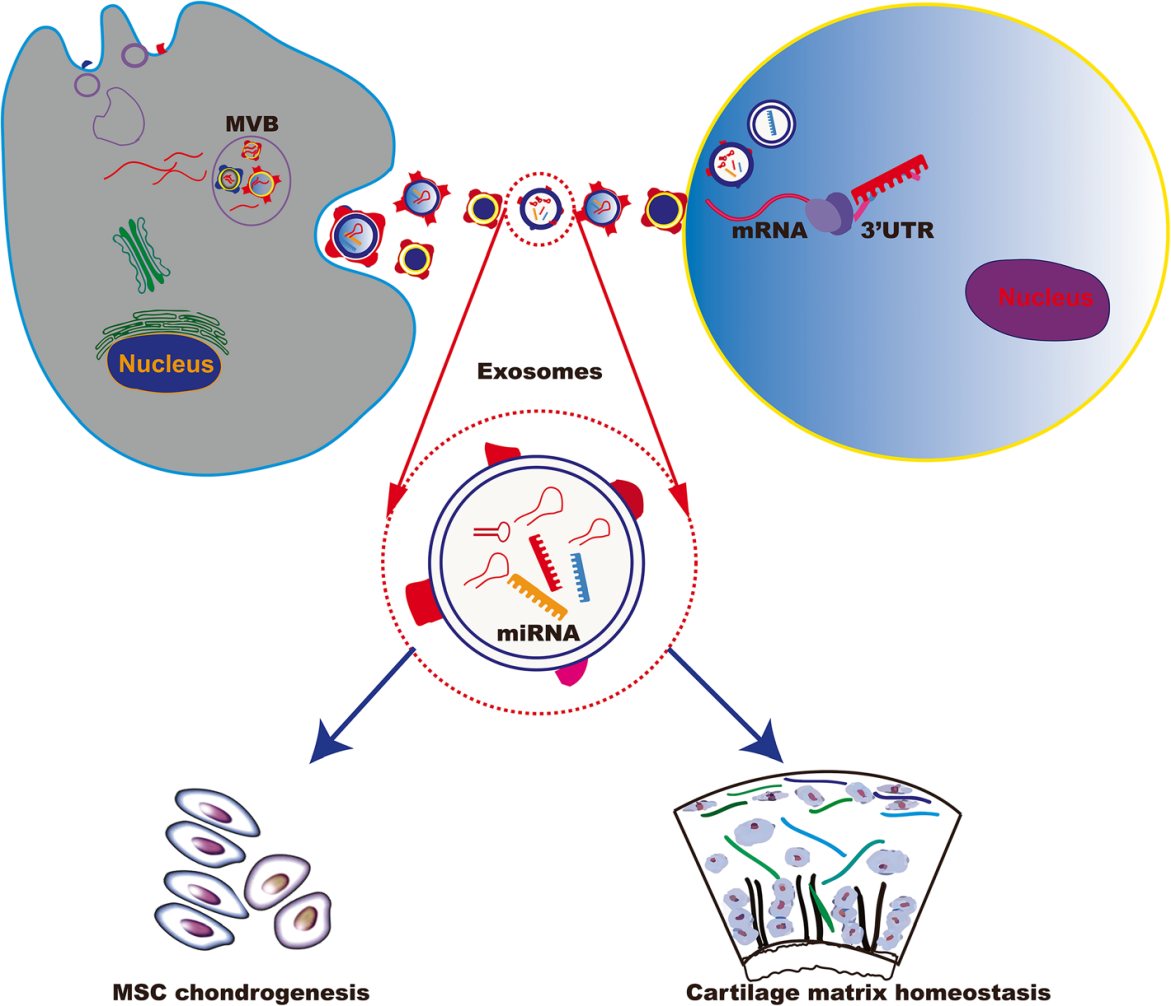
Exosome mediated miR-140 transfer implicated in OA treatment

Viruses are very efficient at infecting human cells, and thus viral vectors are widely used for ex vivo delivery of genes. However, there are several disadvantages of using viral vectors such as oncogenicity from insertional mutations [82]. These disadvantages may compromise the efficacy of gene therapy and limit their application for gene delivery in vivo. Commercially available reagents for miRNA transfection are typical liposomes of micrometre sizes with a broad size distribution. These liposomes can work well on cultured cells, although earlier versions did exhibit some toxicity [83]. Compared to chemical liposomes, natural exosomes for intracellular biomolecule transfer show almost no cell toxicity and have improved storage stability and anti-serum aggregation abilities [84]. Currently, clinical trials of exosomes as cell-free therapy are underway to evaluate their safety and efficacy. Bone marrow stem cells are the typical donor cells of exosomes, and miRNAs are the typical therapeutic cargoes [85]. SF-MSCs have shown higher chondrogenic potential than BM-MSCs [86]. Exosomes purified from autologous SF-MSCs represent exciting new avenues to deliver miRNAs in OA treatment (Fig. 2). Animal studies have demonstrated that exosomes derived from miR-140-5p-overexpressing human SF-MSCs enhance cartilage tissue regeneration and prevent knee OA [68].

Exosomes derived from miR-140-overexpressing cells or miR-140-encapsulated exosomes could be used to promote MSC chondrogenesis or inhibit cartilage matrix degradation for OA therapy.

## Conclusion

Articular cartilage is composed of chondrocytes and cartilage matrix. The dense matrix is synthesized by chondrocytes. miR-140 plays an important role in the stability and maintenance of the cartilage matrix in chondrocytes. Recent advances in epigenetic studies have shown the enormous potential of miR-140 as a therapeutic target for OA. miR-140 can act on different targets to regulate chondrocyte differentiation and OA development at different stages. The effect of miR-140 on chondrocytes and cartilage matrix as well as OA requires deeper investigation and additional experimental research. Concerning miR-140 delivery, exosomes are a promising cargo carrier in OA treatment. In particular, exosomes from autologous SF-MSCs are a safe miR-140 delivery tool without any unwanted immune responses.

However, before consideration as clinical therapeutics, several unanswered aspects of exosomes should be explored: (i) Large-scale amounts of exosomes should be produced to meet clinical requirements. Exosome isolation requires a considerable amount of labour and starting cells, and the preparation should be carefully examined to ensure that its content is consistent over time and devoid of contaminants. (ii) The content of exosomes is cell type- and stimulation-dependent, and given the low amount of available primary cells, the production of exosomes for therapeutic purposes will be limited to exosomes from cell lines, which might not contain the same beneficial exosomal content. (iii) Exosomes contain not only multiple miRNAs but also other biological effectors (proteins, other RNAs, lipids, etc), which might affect the phenotype of uptaking cells. In addition, multiple cell types might uptake these exosomes with unpredictable effects on other tissues. (iv) The methods by which miR-140 is packaged into exosomes should be optimized to maintain high loading efficiency. (v) Cellular metabolism of synthetic miR-140 in vivo should be highly considered. Further studies should be performed to monitor miR-140 function and systematic response after injection into the joint tissue. (vi) Exosomes should be endowed with precise targeting abilities. To accomplish this goal, more precise targeting strategies should be developed. If these challenges are adequately addressed, delivery of miR-140 by exosomes from autologous SF-MSCs will be a reliable and promising method to counteract OA.

## Data Availability

Not applicable.

## Abbreviations

ACAN: Aggrecan
ADAMs: A disintegrin and metalloproteinases
ADAMTS: A disintegrin and metalloproteinase with thrombospondin motifs
BM-MSCs: Bone marrow-derived MSCs
BMP: Bone morphogenic protein
CXCR: C-X-C motif chemokine receptor
C/EBPs: CCAAT/enhancer-binding proteins
COL1: Collagen type I
COL2: Collagen type II
COL3: Collagen type III
COL10: Collagen type X
ECM: Extracellular matrix
ER: Estrogen receptor
HDAC: Histone deacetylase
HMG: High-mobility-group
hUC: Human umbilical cord
IGF1R: Insulin-like growth factor 1 receptor
IGFBP: Insulin-like growth factor-binding protein
IL-1β: Interleukin-1 beta
JAG1: Jagged1
miRNAs: MicroRNAs
miR-140: MicroRNA-140
MMP: Matrix metalloproteinases
mRNA: Messenger RNA
MSCs: Mesenchymal stem cells
NUMBL: NUMB-like endocytic adaptor protein
OA: Osteoarthritis
PI3K: Phosphatidylinositol 3′-kinase
RALA: RAS-like proto-oncogene A
SF: Synovial fluid
SMAD: Mothers against decapentaplegic homologue
SOX: SRY-related high-mobility group box
TGFBR: Transforming growth factor beta receptor
TLR: Toll-like receptor
TNF-α: Tumour necrosis factor-alpha
UTR: Untranslated region
WWP2: WW domain-containing E3 ubiquitin-protein ligase 2
